# *N*,*N*′-Dicyclo­hexyl-*N*′′-(2,6-difluoro­benzo­yl)-*N*,*N*′-dimethyl­phospho­ric triamide

**DOI:** 10.1107/S1600536811043029

**Published:** 2011-10-22

**Authors:** Mehrdad Pourayoubi, Atekeh Tarahhomi, Arnold L. Rheingold, James A. Golen

**Affiliations:** a Department of Chemistry, Ferdowsi University of Mashhad, Mashhad 91779, Iran; b Department of Chemistry, University of California, San Diego, 9500 Gilman Drive, La Jolla, CA 92093, USA

## Abstract

In the title mol­ecule, C_21_H_32_F_2_N_3_O_2_P, the P=O and N—H groups are *syn* with respect to each other, and the P atom is bonded in a distorted tetra­hedral environment. The phosphoryl group adopts an *anti* orientation with respect to the carbonyl group. The angles at the tertiary N atoms (with bond-angle sums of 358.4 and 357.0°) confirm their *sp* ^2^ character. In the crystal, inversion dimers linked by pairs of N—H⋯O hydrogen bonds generate *R*_2_^2^(8) loops.

## Related literature

For hydrogen-bond patterns in compounds containing a C(O)NHP(O) skeleton, see: Toghraee *et al.* (2011 ▶); Pourayoubi *et al.* (2011 ▶). For background to phospho­ric triamide compounds containing a C(O)NHP(O) skeleton, and related bond lengths, angles and torsion angles, see: Pourayoubi *et al.* (2010 ▶); Amirkhanov *et al.* (2010 ▶); Tarahhomi *et al.* (2011 ▶). For a description of hydrogen-bond motifs, see: Bernstein *et al.* (1995 ▶).
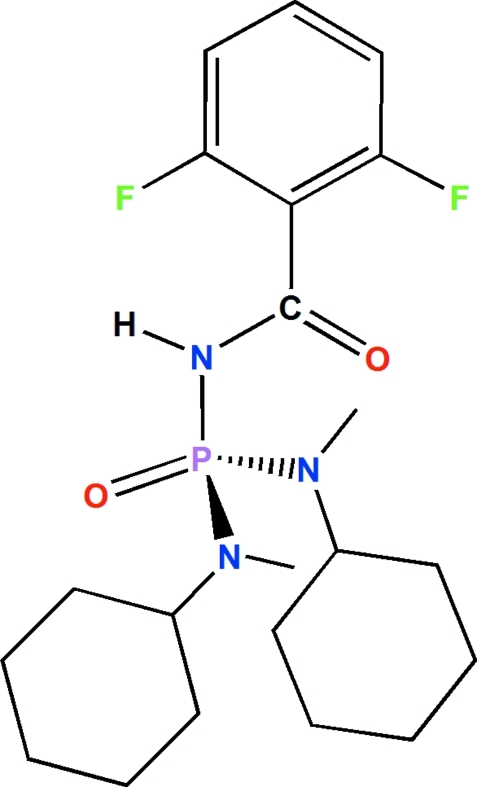


## Experimental

### 

#### Crystal data

C_21_H_32_F_2_N_3_O_2_P
*M* *_r_* = 427.47
Triclinic, 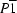

*a* = 10.2322 (6) Å
*b* = 10.6188 (5) Å
*c* = 11.2658 (6) Å
α = 69.419 (1)°
β = 79.269 (1)°
γ = 81.536 (1)°
*V* = 1121.45 (10) Å^3^
*Z* = 2
Mo *K*α radiation
μ = 0.16 mm^−1^
*T* = 100 K
0.35 × 0.30 × 0.25 mm

#### Data collection

Bruker SMART CCD area-detector diffractometer
Absorption correction: multi-scan (*SADABS*; Bruker, 2005 ▶) *T*_min_ = 0.946, *T*_max_ = 0.961
17147 measured reflections
5053 independent reflections
4421 reflections with *I* > 2σ(*I*)
*R*_int_ = 0.029

#### Refinement

*R*[*F* ^2^ > 2σ(*F* ^2^)] = 0.037
*wR*(*F* ^2^) = 0.097
*S* = 1.05
5053 reflections
267 parameters
1 restraint
H atoms treated by a mixture of independent and constrained refinement
Δρ_max_ = 0.32 e Å^−3^
Δρ_min_ = −0.34 e Å^−3^

### 

Data collection: *SMART* (Bruker, 2005 ▶); cell refinement: *SAINT* (Bruker, 2005 ▶); data reduction: *SAINT*; program(s) used to solve structure: *SHELXS97* (Sheldrick, 2008 ▶); program(s) used to refine structure: *SHELXL97* (Sheldrick, 2008 ▶); molecular graphics: *SHELXTL* (Sheldrick, 2008 ▶); software used to prepare material for publication: *SHELXTL* and *enCIFer* (Allen *et al.*, 2004 ▶).

## Supplementary Material

Crystal structure: contains datablock(s) I, global. DOI: 10.1107/S1600536811043029/bh2384sup1.cif

Structure factors: contains datablock(s) I. DOI: 10.1107/S1600536811043029/bh2384Isup2.hkl

Additional supplementary materials:  crystallographic information; 3D view; checkCIF report

## Figures and Tables

**Table 1:** Hydrogen-bond geometry (Å, °)

*D*—H⋯*A*	*D*—H	H⋯*A*	*D*⋯*A*	*D*—H⋯*A*
N1—H1N⋯O2^i^	0.86 (1)	1.90 (1)	2.7330 (13)	165 (1)

Symmetry code: (i) 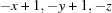.

**Fig. 1.**
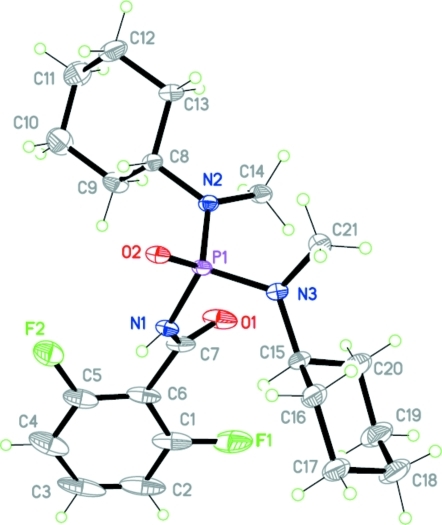
An ORTEP-style plot for the title compound. Displacement ellipsoids are given at 50% probability level and H atoms are drawn as small spheres of arbitrary radii.

## supplementary crystallographic information

### Comment

The patterns of hydrogen bonds and their strengths on phosphoric triamides containing a C(O)NHP(O) skeleton have been discussed (Toghraee *et al.*, 2011; Pourayoubi *et al.*, 2011). The structure determination of the title compound, P(O)[2,6-F_2_—C_6_H_3_C(O)NH][N(CH_3_)C_6_H_11_]_2_ (Fig. 1), was performed as part of a project in our laboratory on the synthesis of new derivatives of benzoyl phosphoric triamides with formula *X*C_6_H*_n_*C(O)NHP(O)*Y*_2_ (for example, *n* = 3, *X* = 2,6-F_2_ and *Y* = NHC(CH_3_)_3_: Pourayoubi *et al.*, 2010).

In the title phosphoric triamide, the phosphoryl group adopts the *anti* orientation with respect to the carbonyl group; whereas it is in a *syn* position relative to the N—H unit. The tetrahedral environment at the P atom is distorted, as previously noted for other phosphoric triamides (Amirkhanov *et al.*, 2010). The P═O, C═O and P—N bond lengths are within the expected ranges (Tarahhomi *et al.*, 2011).

The hydrogen atom of the C(═O)NHP(═O) moiety is involved in an intermolecular –P═O···H—N– hydrogen bond. Pairs of this type of hydrogen bonds form hydrogen-bonded dimers in the crystal (as *R*_2_^2^(8) rings; see: Bernstein *et al.*, 1995).

### Experimental

2,6—F_2_C_6_H_3_C(O)NHP(O)Cl_2_ was prepared according to the literature method reported by Pourayoubi *et al.* (2010).

The title compound was synthesized from the reaction of 2,6—F_2_C_6_H_3_C(O)NHP(O)Cl_2_ (1.09 mmol) with *N*-methylcyclohexylamine (4.36 mmol) in dry chloroform (30 ml). The amine was added dropwise to a solution of 2,6—F_2_C_6_H_3_C(O)NHP(O)Cl_2_ at 273 K, with continuous stirring. After 4 h, the solvent was evaporated and the obtained solid was washed with distilled water and recrystallized from a mixture of chloroform and DMF (4:1 *v*/*v*) at room temperature. IR (KBr, ν, cm^-1^): 3063 (NH), 2912, 2752, 1698, 1608, 1470, 1262, 1192, 1155, 997, 864, 815.

### Refinement

All non-H atoms were refined anisotropically by full-matrix least-squares on *F*^2^. Hydrogen H1N was found in a difference map and N1—H1N distance was set at 0.87 (1) Å and allowed to refine with *U*_iso_ = 1.2*U*_eq_(N1). All other H atoms were placed in calculated positions with C—H distances for CH_2_ of 0.99 Å, CH_3_ of 0.98 Å, methine CH of 1.00 Å, and C(Ar)H 0.95 Å, and with *U*_iso_ of 1.2 or 1.5 times that of the parent C atom.

### Crystal data

**Table:**

C_21_H_32_F_2_N_3_O_2_P	*Z* = 2
*M**_r_* = 427.47	*F*(000) = 456
Triclinic, *P*1	*D*_x_ = 1.266 Mg m^−^^3^
Hall symbol: -P 1	Mo *K*α radiation, λ = 0.71073 Å
*a* = 10.2322 (6) Å	Cell parameters from 9954 reflections
*b* = 10.6188 (5) Å	θ = 2.4–28.0°
*c* = 11.2658 (6) Å	µ = 0.16 mm^−^^1^
α = 69.419 (1)°	*T* = 100 K
β = 79.269 (1)°	Block, colourless
γ = 81.536 (1)°	0.35 × 0.30 × 0.25 mm
*V* = 1121.45 (10) Å^3^	

### Data collection

**Table:**

Bruker SMART CCD area-detector diffractometer	5053 independent reflections
Radiation source: fine-focus sealed tube	4421 reflections with *I* > 2σ(*I*)
graphite	*R*_int_ = 0.029
φ and ω scans	θ_max_ = 28.0°, θ_min_ = 2.0°
Absorption correction: multi-scan (*SADABS*; Bruker, 2005)	*h* = −13→13
*T*_min_ = 0.946, *T*_max_ = 0.961	*k* = −13→13
17147 measured reflections	*l* = −14→14

### Refinement

**Table:**

Refinement on *F*^2^	Primary atom site location: structure-invariant direct methods
Least-squares matrix: full	Secondary atom site location: difference Fourier map
*R*[*F*^2^ > 2σ(*F*^2^)] = 0.037	Hydrogen site location: inferred from neighbouring sites
*wR*(*F*^2^) = 0.097	H atoms treated by a mixture of independent and constrained refinement
*S* = 1.05	*w* = 1/[σ^2^(*F*_o_^2^) + (0.0429*P*)^2^ + 0.4607*P*] where *P* = (*F*_o_^2^ + 2*F*_c_^2^)/3
5053 reflections	(Δ/σ)_max_ < 0.001
267 parameters	Δρ_max_ = 0.32 e Å^−^^3^
1 restraint	Δρ_min_ = −0.34 e Å^−^^3^
0 constraints	

### Fractional atomic coordinates and isotropic or equivalent isotropic displacement parameters (Å^2^)

**Table:**

	*x*	*y*	*z*	*U*_iso_*/*U*_eq_	
P1	0.42370 (3)	0.41644 (3)	0.20824 (3)	0.01482 (9)	
F1	0.91928 (9)	0.33633 (12)	0.27902 (10)	0.0491 (3)	
F2	0.68054 (9)	0.73518 (9)	0.04970 (9)	0.0363 (2)	
O1	0.64907 (10)	0.41510 (13)	0.35266 (9)	0.0347 (3)	
O2	0.35023 (8)	0.47928 (9)	0.09668 (8)	0.01753 (19)	
N1	0.57558 (10)	0.47579 (11)	0.15972 (10)	0.0170 (2)	
H1N	0.5985 (14)	0.5054 (14)	0.0781 (9)	0.020*	
N2	0.34278 (10)	0.45423 (10)	0.33252 (9)	0.0165 (2)	
N3	0.45145 (11)	0.25173 (11)	0.25782 (11)	0.0244 (2)	
C1	0.91678 (14)	0.4643 (2)	0.19652 (16)	0.0392 (4)	
C2	1.03613 (16)	0.5176 (3)	0.14059 (19)	0.0497 (5)	
H2	1.1182	0.4669	0.1619	0.060*	
C3	1.03445 (16)	0.6460 (3)	0.0529 (2)	0.0546 (6)	
H3	1.1166	0.6842	0.0137	0.066*	
C4	0.91532 (17)	0.7212 (2)	0.02025 (17)	0.0463 (5)	
H4	0.9148	0.8099	−0.0405	0.056*	
C5	0.79751 (14)	0.66235 (18)	0.07925 (15)	0.0333 (4)	
C6	0.79311 (13)	0.53390 (17)	0.16838 (13)	0.0283 (3)	
C7	0.66617 (13)	0.46961 (15)	0.23652 (13)	0.0236 (3)	
C8	0.27329 (12)	0.59130 (12)	0.31148 (11)	0.0169 (2)	
H8	0.2504	0.6255	0.2224	0.020*	
C9	0.36202 (14)	0.69035 (14)	0.32103 (14)	0.0258 (3)	
H9A	0.3890	0.6578	0.4075	0.031*	
H9B	0.4439	0.6951	0.2575	0.031*	
C10	0.28793 (18)	0.83088 (16)	0.29625 (17)	0.0388 (4)	
H10A	0.3451	0.8918	0.3081	0.047*	
H10B	0.2701	0.8673	0.2064	0.047*	
C11	0.15626 (17)	0.82817 (16)	0.38598 (17)	0.0385 (4)	
H11A	0.1743	0.8016	0.4753	0.046*	
H11B	0.1085	0.9197	0.3635	0.046*	
C12	0.06889 (15)	0.72885 (15)	0.37619 (16)	0.0328 (3)	
H12A	0.0434	0.7605	0.2892	0.039*	
H12B	−0.0139	0.7250	0.4385	0.039*	
C13	0.14254 (13)	0.58770 (14)	0.40322 (13)	0.0222 (3)	
H13A	0.1616	0.5529	0.4927	0.027*	
H13B	0.0852	0.5257	0.3933	0.027*	
C14	0.36037 (14)	0.37310 (14)	0.46557 (12)	0.0248 (3)	
H14A	0.3893	0.4297	0.5066	0.037*	
H14B	0.4281	0.2975	0.4658	0.037*	
H14C	0.2754	0.3381	0.5129	0.037*	
C15	0.57908 (14)	0.17673 (14)	0.22547 (14)	0.0277 (3)	
H15	0.6473	0.2439	0.1874	0.033*	
C16	0.57337 (16)	0.11177 (15)	0.12540 (14)	0.0335 (3)	
H16A	0.5479	0.1824	0.0465	0.040*	
H16B	0.5042	0.0468	0.1585	0.040*	
C17	0.70838 (18)	0.03820 (16)	0.09313 (15)	0.0400 (4)	
H17A	0.6998	−0.0088	0.0334	0.048*	
H17B	0.7750	0.1049	0.0497	0.048*	
C18	0.7570 (2)	−0.06431 (18)	0.21368 (16)	0.0476 (5)	
H18A	0.6957	−0.1369	0.2517	0.057*	
H18B	0.8468	−0.1059	0.1906	0.057*	
C19	0.76312 (19)	0.00220 (19)	0.31179 (17)	0.0465 (5)	
H19A	0.8303	0.0692	0.2766	0.056*	
H19B	0.7914	−0.0671	0.3901	0.056*	
C20	0.62703 (17)	0.07244 (16)	0.34615 (15)	0.0362 (4)	
H20A	0.6341	0.1178	0.4076	0.043*	
H20B	0.5613	0.0045	0.3877	0.043*	
C21	0.33158 (15)	0.17706 (14)	0.30012 (15)	0.0304 (3)	
H21A	0.3101	0.1560	0.2283	0.046*	
H21B	0.2564	0.2325	0.3300	0.046*	
H21C	0.3483	0.0930	0.3702	0.046*	

### Atomic displacement parameters (Å^2^)

**Table:**

	*U*^11^	*U*^22^	*U*^33^	*U*^12^	*U*^13^	*U*^23^
P1	0.01077 (15)	0.01879 (16)	0.01385 (15)	0.00019 (11)	−0.00010 (11)	−0.00559 (12)
F1	0.0221 (5)	0.0863 (8)	0.0401 (6)	0.0158 (5)	−0.0113 (4)	−0.0274 (6)
F2	0.0281 (5)	0.0433 (5)	0.0395 (5)	−0.0109 (4)	0.0008 (4)	−0.0161 (4)
O1	0.0187 (5)	0.0684 (8)	0.0170 (5)	0.0035 (5)	−0.0043 (4)	−0.0164 (5)
O2	0.0119 (4)	0.0264 (5)	0.0154 (4)	−0.0013 (3)	−0.0006 (3)	−0.0092 (4)
N1	0.0116 (5)	0.0268 (5)	0.0126 (5)	−0.0012 (4)	−0.0006 (4)	−0.0074 (4)
N2	0.0140 (5)	0.0206 (5)	0.0124 (5)	0.0026 (4)	−0.0013 (4)	−0.0044 (4)
N3	0.0186 (6)	0.0198 (5)	0.0281 (6)	0.0026 (4)	0.0060 (5)	−0.0061 (5)
C1	0.0152 (7)	0.0836 (13)	0.0351 (8)	0.0013 (7)	−0.0044 (6)	−0.0418 (9)
C2	0.0152 (7)	0.1089 (17)	0.0493 (11)	−0.0083 (9)	0.0001 (7)	−0.0576 (12)
C3	0.0188 (8)	0.1161 (19)	0.0591 (12)	−0.0293 (10)	0.0157 (8)	−0.0679 (13)
C4	0.0354 (9)	0.0766 (13)	0.0434 (10)	−0.0298 (9)	0.0141 (7)	−0.0405 (10)
C5	0.0183 (7)	0.0595 (10)	0.0340 (8)	−0.0111 (7)	0.0033 (6)	−0.0307 (8)
C6	0.0125 (6)	0.0588 (10)	0.0244 (7)	−0.0046 (6)	−0.0005 (5)	−0.0274 (7)
C7	0.0127 (6)	0.0419 (8)	0.0202 (6)	0.0028 (5)	−0.0026 (5)	−0.0170 (6)
C8	0.0153 (6)	0.0198 (6)	0.0155 (6)	0.0015 (5)	−0.0024 (4)	−0.0070 (5)
C9	0.0236 (7)	0.0311 (7)	0.0262 (7)	−0.0076 (6)	0.0032 (5)	−0.0154 (6)
C10	0.0485 (10)	0.0270 (8)	0.0419 (9)	−0.0089 (7)	0.0094 (8)	−0.0186 (7)
C11	0.0439 (10)	0.0299 (8)	0.0419 (9)	0.0036 (7)	0.0046 (7)	−0.0202 (7)
C12	0.0257 (8)	0.0348 (8)	0.0367 (8)	0.0103 (6)	−0.0032 (6)	−0.0163 (7)
C13	0.0143 (6)	0.0279 (7)	0.0246 (7)	0.0012 (5)	0.0004 (5)	−0.0120 (5)
C14	0.0206 (7)	0.0320 (7)	0.0141 (6)	0.0053 (5)	−0.0007 (5)	−0.0023 (5)
C15	0.0244 (7)	0.0221 (7)	0.0261 (7)	0.0086 (5)	0.0052 (6)	−0.0041 (5)
C16	0.0380 (9)	0.0265 (7)	0.0252 (7)	0.0099 (6)	0.0039 (6)	−0.0049 (6)
C17	0.0461 (10)	0.0314 (8)	0.0272 (8)	0.0161 (7)	0.0073 (7)	−0.0051 (6)
C18	0.0545 (12)	0.0374 (9)	0.0338 (9)	0.0271 (8)	0.0017 (8)	−0.0075 (7)
C19	0.0461 (11)	0.0447 (10)	0.0351 (9)	0.0269 (8)	−0.0058 (8)	−0.0087 (7)
C20	0.0383 (9)	0.0343 (8)	0.0266 (8)	0.0154 (7)	−0.0009 (7)	−0.0080 (6)
C21	0.0281 (8)	0.0204 (7)	0.0364 (8)	−0.0035 (5)	0.0085 (6)	−0.0078 (6)

### Geometric parameters (Å, °)

**Table:**

P1—O2	1.4848 (9)	C11—C12	1.523 (2)
P1—N2	1.6348 (11)	C11—H11A	0.9900
P1—N3	1.6363 (11)	C11—H11B	0.9900
P1—N1	1.6854 (10)	C12—C13	1.5295 (19)
F1—C1	1.350 (2)	C12—H12A	0.9900
F2—C5	1.3552 (19)	C12—H12B	0.9900
O1—C7	1.2205 (16)	C13—H13A	0.9900
N1—C7	1.3612 (15)	C13—H13B	0.9900
N1—H1N	0.859 (9)	C14—H14A	0.9800
N2—C14	1.4725 (15)	C14—H14B	0.9800
N2—C8	1.4819 (15)	C14—H14C	0.9800
N3—C21	1.4707 (17)	C15—C16	1.528 (2)
N3—C15	1.4810 (17)	C15—C20	1.5314 (19)
C1—C2	1.369 (2)	C15—H15	1.0000
C1—C6	1.399 (2)	C16—C17	1.532 (2)
C2—C3	1.374 (3)	C16—H16A	0.9900
C2—H2	0.9500	C16—H16B	0.9900
C3—C4	1.390 (3)	C17—C18	1.526 (2)
C3—H3	0.9500	C17—H17A	0.9900
C4—C5	1.384 (2)	C17—H17B	0.9900
C4—H4	0.9500	C18—C19	1.521 (3)
C5—C6	1.382 (2)	C18—H18A	0.9900
C6—C7	1.5046 (18)	C18—H18B	0.9900
C8—C13	1.5271 (17)	C19—C20	1.530 (2)
C8—C9	1.5294 (17)	C19—H19A	0.9900
C8—H8	1.0000	C19—H19B	0.9900
C9—C10	1.527 (2)	C20—H20A	0.9900
C9—H9A	0.9900	C20—H20B	0.9900
C9—H9B	0.9900	C21—H21A	0.9800
C10—C11	1.524 (2)	C21—H21B	0.9800
C10—H10A	0.9900	C21—H21C	0.9800
C10—H10B	0.9900		
			
O2—P1—N2	110.40 (5)	C11—C12—C13	110.95 (12)
O2—P1—N3	116.97 (6)	C11—C12—H12A	109.5
N2—P1—N3	105.60 (6)	C13—C12—H12A	109.5
O2—P1—N1	105.57 (5)	C11—C12—H12B	109.5
N2—P1—N1	112.66 (5)	C13—C12—H12B	109.5
N3—P1—N1	105.73 (6)	H12A—C12—H12B	108.0
C7—N1—P1	126.27 (9)	C8—C13—C12	110.68 (11)
C7—N1—H1N	118.7 (10)	C8—C13—H13A	109.5
P1—N1—H1N	114.7 (10)	C12—C13—H13A	109.5
C14—N2—C8	116.58 (10)	C8—C13—H13B	109.5
C14—N2—P1	123.33 (9)	C12—C13—H13B	109.5
C8—N2—P1	118.49 (8)	H13A—C13—H13B	108.1
C21—N3—C15	116.90 (11)	N2—C14—H14A	109.5
C21—N3—P1	115.51 (9)	N2—C14—H14B	109.5
C15—N3—P1	124.57 (9)	H14A—C14—H14B	109.5
F1—C1—C2	118.10 (16)	N2—C14—H14C	109.5
F1—C1—C6	118.56 (14)	H14A—C14—H14C	109.5
C2—C1—C6	123.28 (19)	H14B—C14—H14C	109.5
C1—C2—C3	118.40 (17)	N3—C15—C16	113.22 (12)
C1—C2—H2	120.8	N3—C15—C20	111.07 (11)
C3—C2—H2	120.8	C16—C15—C20	111.21 (12)
C2—C3—C4	121.52 (15)	N3—C15—H15	107.0
C2—C3—H3	119.2	C16—C15—H15	107.0
C4—C3—H3	119.2	C20—C15—H15	107.0
C5—C4—C3	117.7 (2)	C15—C16—C17	111.21 (14)
C5—C4—H4	121.1	C15—C16—H16A	109.4
C3—C4—H4	121.1	C17—C16—H16A	109.4
F2—C5—C6	118.33 (12)	C15—C16—H16B	109.4
F2—C5—C4	118.37 (17)	C17—C16—H16B	109.4
C6—C5—C4	123.29 (16)	H16A—C16—H16B	108.0
C5—C6—C1	115.79 (14)	C18—C17—C16	111.24 (13)
C5—C6—C7	124.16 (13)	C18—C17—H17A	109.4
C1—C6—C7	120.02 (15)	C16—C17—H17A	109.4
O1—C7—N1	123.71 (12)	C18—C17—H17B	109.4
O1—C7—C6	120.91 (11)	C16—C17—H17B	109.4
N1—C7—C6	115.38 (11)	H17A—C17—H17B	108.0
N2—C8—C13	111.30 (10)	C19—C18—C17	111.16 (14)
N2—C8—C9	112.22 (10)	C19—C18—H18A	109.4
C13—C8—C9	110.75 (10)	C17—C18—H18A	109.4
N2—C8—H8	107.4	C19—C18—H18B	109.4
C13—C8—H8	107.4	C17—C18—H18B	109.4
C9—C8—H8	107.4	H18A—C18—H18B	108.0
C10—C9—C8	110.65 (12)	C18—C19—C20	110.98 (16)
C10—C9—H9A	109.5	C18—C19—H19A	109.4
C8—C9—H9A	109.5	C20—C19—H19A	109.4
C10—C9—H9B	109.5	C18—C19—H19B	109.4
C8—C9—H9B	109.5	C20—C19—H19B	109.4
H9A—C9—H9B	108.1	H19A—C19—H19B	108.0
C11—C10—C9	111.80 (13)	C19—C20—C15	110.31 (12)
C11—C10—H10A	109.3	C19—C20—H20A	109.6
C9—C10—H10A	109.3	C15—C20—H20A	109.6
C11—C10—H10B	109.3	C19—C20—H20B	109.6
C9—C10—H10B	109.3	C15—C20—H20B	109.6
H10A—C10—H10B	107.9	H20A—C20—H20B	108.1
C12—C11—C10	110.74 (13)	N3—C21—H21A	109.5
C12—C11—H11A	109.5	N3—C21—H21B	109.5
C10—C11—H11A	109.5	H21A—C21—H21B	109.5
C12—C11—H11B	109.5	N3—C21—H21C	109.5
C10—C11—H11B	109.5	H21A—C21—H21C	109.5
H11A—C11—H11B	108.1	H21B—C21—H21C	109.5
			
O2—P1—N1—C7	164.83 (11)	P1—N1—C7—C6	−178.43 (10)
N2—P1—N1—C7	44.27 (13)	C5—C6—C7—O1	−131.18 (15)
N3—P1—N1—C7	−70.59 (12)	C1—C6—C7—O1	47.0 (2)
O2—P1—N2—C14	157.51 (10)	C5—C6—C7—N1	49.58 (19)
N3—P1—N2—C14	30.20 (11)	C1—C6—C7—N1	−132.22 (14)
N1—P1—N2—C14	−84.74 (11)	C14—N2—C8—C13	−50.03 (14)
O2—P1—N2—C8	−37.41 (10)	P1—N2—C8—C13	143.89 (9)
N3—P1—N2—C8	−164.72 (9)	C14—N2—C8—C9	74.73 (13)
N1—P1—N2—C8	80.35 (9)	P1—N2—C8—C9	−91.36 (11)
O2—P1—N3—C21	−60.31 (12)	N2—C8—C9—C10	178.90 (11)
N2—P1—N3—C21	62.93 (11)	C13—C8—C9—C10	−56.04 (15)
N1—P1—N3—C21	−177.45 (10)	C8—C9—C10—C11	55.54 (17)
O2—P1—N3—C15	99.32 (12)	C9—C10—C11—C12	−55.57 (19)
N2—P1—N3—C15	−137.44 (11)	C10—C11—C12—C13	56.09 (18)
N1—P1—N3—C15	−17.82 (13)	N2—C8—C13—C12	−177.37 (10)
F1—C1—C2—C3	177.59 (14)	C9—C8—C13—C12	57.05 (14)
C6—C1—C2—C3	0.3 (2)	C11—C12—C13—C8	−57.15 (16)
C1—C2—C3—C4	−0.3 (2)	C21—N3—C15—C16	54.69 (16)
C2—C3—C4—C5	0.0 (2)	P1—N3—C15—C16	−104.68 (13)
C3—C4—C5—F2	179.09 (13)	C21—N3—C15—C20	−71.27 (17)
C3—C4—C5—C6	0.2 (2)	P1—N3—C15—C20	129.36 (12)
F2—C5—C6—C1	−179.04 (12)	N3—C15—C16—C17	178.75 (11)
C4—C5—C6—C1	−0.2 (2)	C20—C15—C16—C17	−55.36 (16)
F2—C5—C6—C7	−0.8 (2)	C15—C16—C17—C18	54.61 (19)
C4—C5—C6—C7	178.10 (13)	C16—C17—C18—C19	−55.4 (2)
F1—C1—C6—C5	−177.37 (12)	C17—C18—C19—C20	56.9 (2)
C2—C1—C6—C5	−0.1 (2)	C18—C19—C20—C15	−57.2 (2)
F1—C1—C6—C7	4.3 (2)	N3—C15—C20—C19	−176.48 (14)
C2—C1—C6—C7	−178.47 (13)	C16—C15—C20—C19	56.45 (18)
P1—N1—C7—O1	2.4 (2)		

### Hydrogen-bond geometry (Å, °)

**Table:**

*D*—H···*A*	*D*—H	H···*A*	*D*···*A*	*D*—H···*A*
N1—H1N···O2^i^	0.86 (1)	1.90 (1)	2.7330 (13)	165.(1)

Symmetry codes: (i) −*x*+1, −*y*+1, −*z*.
